# The Bodies “at the Forefront”: Mentalization, Memory, and Construction of the Self during Adolescence

**DOI:** 10.3389/fpsyg.2017.01502

**Published:** 2017-09-01

**Authors:** Antonella Marchetti, Davide Massaro, Cinzia Di Dio

**Affiliations:** Department of Psychology, Università Cattolica del Sacro Cuore Milan, Italy

**Keywords:** mentalization, memory, body, adolescence, self-identity, narrative

With this contribution, we offer a perspective focused on the mind-body relation in a specific phase of the life span: adolescence. In particular, we look at the complexity of some of the processes involved in the construction of an adult Self, which results from the interaction between experiences from infancy and a changing body during adolescence accompanied by implemented mental and social abilities/possibilities. Our interpretative hypothesis goes back to the construct of mentalization, focusing on the function of implicit bodily memories entwined with infantile experiences as precursors of relational dynamics that are mentalistically mediated. When troubled, these need to be dealt with, particularly during adolescence, when the demanding and inedited quests of the body may represent a hurdle that has to be overcome to achieve the formation of an integrated identity.

The development of cognition, the complexity of behavioral and social processes and, not last, sexual maturation mark adolescence (e.g., Camaioni and Di Blasio, 2007; Faliva and Cozzani, 2011), defined as period “in transition” (Rutter, 1992; Palmonari, 1997). The individual develops a sense of self from infancy; however, during adolescence the very first conscious effort is made to answer to the question “Who am I?.” This involves the organization of the individual's abilities, beliefs and history with the aim to form a coherent image of the Self through a sense of continuity over time (Pasupathi and Hoyt, 2009). In this stage, beliefs about the Self as well as beliefs of others are integrated within the process of identity development, which requires reaching a balance between commitment and confusion about one's beliefs, goals, values, and roles in society (Erikson, 1963). When commitment occurs, possibly after exploration of different alternatives, the adolescent can effectively resolve the identity issue dodging a chance to remain in a state of “identity diffusion” characterized by absence of integration and lack of commitment (Makros and McCabe, 2001). This scenario is further challenged by changes occurring in the adolescent's social cognitive and emotional brain, which significantly affect social behavior and choices during adolescence and, namely, the process of “social reorientation” (Nelson et al., 2005; see also Immordino-Yang, 2016).

Within this complex frame, adolescents are conventionally thought to build their identity hinging psychic with social (e.g., Erikson, 1950, 1953), very much in line with Mead's general conception of Self-construction (Mead, 1913). Less attention has been paid to maturation of the body, the first biological marker of our lifelong experiences. In fact, as thoroughly argued by several authors below discussed, in the transition from primary to tertiary intersubjectivity, the individual's transforming body and brain grow in active engagement with an environment of human factors—organic at first, then psychological or inter-mental (Trevarthen and Hubley, 1978; Trevarthen and Aitken, 2001; see also, Stern, 1985, 2004; Seganti, 1995).

Let's try then to reverse the classical view placing the body at the “forefront.” The body is from birth (and even from the uterus) a constructive and expressive vehicle of the Self. Not by chance, it has been suggested to add the body to the four levels of Doise's psychosocial analysis (Doise, 1982, Doise and Mapstone, 1986; Brunel and Cosnier, 2012). Then, how do we enquire the body to support the construction of an identity, the developmental transition from infancy to subsequent epochs, ferrying through adolescence? A plausible approach is to look at what happens when things “go wrong.” It is emblematic that adolescents choose the body to express their discomfort: the developmental *impasse* in shaping the Self is explicit through injury of one's own body that—even if within dynamics involving different etiopathogenetic factors—represents a glaring example of how ruptures in developmental milestones carry effects through from infancy to childhood, ultimately reflecting on the adolescents' incapacity to manage all the dynamics underpinning their balanced maturation (Sempio Liverta et al., 2005; Midgley and Vrouva, 2012; Marchetti and Cavalli, 2013; Marchetti et al., 2013). In this respect, the capacity to *mentalize* represents a critical forerunner of the teen' perception of the physical Self, as well as of decisions taken with respect to their body. During the child's development, mentalization implies a gradual acquisition of the ability to correctly attribute mental states to oneself and others to understand behaviors, a maturation of reflective processes regarding people's internal states (Fonagy and Target, 2003; Allen and Fonagy, 2006). Mentalization is a construct that primarily origins from clinical studies and that—more than the construct of Theory of Mind, which mainly refers to an ability—implies one's *propensity* to look at social events in terms of mental states. It follows that, wherever mentalization fails, repercussions of different types and degrees can be observed affecting the child's behavior before, and the adolescent's psychological and physical integrity after, in a continuum between typical and atypical development.

Now, how can we approach mentalization failures that, as hypothesized initially, may be also tied to implicit, bodily rooted, memories shaped by infantile experiences? Moreover, assuming these dynamics entrenched in the adolescent's current perception of the Self, how can the teen's identification process be redirected and reinstated?

To leave something behind, it needs to be somehow remembered; to leave infancy, one needs to be able to disclose it. One of the most frequent and powerful human activities in which this process can take place is narrative: there seems to be no other way of “describing “lived time” save in the form of a narrative,” a selective achievement of memory recall (Bruner, 2004, p. 692; see also Ricoeur, 1981; Nelson, 1989). For Bruner, narrative - as approached from a constructivist view intended as the mind “making the world”—encloses the sense of Self, one's autobiographical history (Bruner, 2004), which inevitably binds to who we are here and now and to what we carry around from our culture (see, e.g., Bruner, 1987, 1991, 2014). Relating mentalization and narrative, years of research on narrative (e.g., Feeney et al., 1994; Fonagy et al., 1998; see also, Van IJzendoorn and Bakermans-Kranenburg, 2008; George and West, 2012; Rossouw and Fonagy, 2012) emphasize on the idea that early experiences with primary caregivers are internalized and eventually reflected in the adults' self-narratives (Waters et al., 2017), which are to be viewed not as a record of what “actually happened” but rather as a continuing reinterpretation of one's experiences (Bruner, 2004). Seen from this perspective, one can then muster the use of memory through narration—regarded in this light as mentalization means—to build a sense of continuity against threats of identity fragmentation. Narrative-based practices would then emerge as an effective strategy helping teenagers found on infantile narratives an “upgraded” narrative accounting for the new personal resources and social quests. It is clear that this process does not imply denying or forgetting the past identity, but rather its integration—within one's own personality—with the new emerging identity.

But what narrative and which forms of memory are to be used that will not betray what said here at the beginning, and namely that the bridge that the mind needs to cross to reach the adult land is the body? From very early in life, the child is seen as an individual able to continuously self-regulate (e.g., Tronick, 1982, 1989; Stern, 1985). A cognitive-affective system of attachment emerges, able to continuously monitor the surrounding environment and endowing the child with procedural knowledge of his internal states with respect to relational events. These procedures incorporate, according to the “sensory memory of attachment relationships” (Seganti, 1995), information regarding the body recorded as sequences of activation states. Any attempt by the child to establish and maintain a balance between his/her internal system and the interaction with the caregiver would determine behavioral modifications persisting in memory (Tronick, 1989) that translate, in the adult life, in a bodily “unconscious/intuitive” response to the environmental stimuli (Seganti, 1995).

Also Stern (1985, 2000, 2004) supported the idea that the transmodal diffusion of the activation levels persist in the adult as a continuous source of extra-linguistic, non-verbal, evaluation of the interactive states with the others, which unfolds in parallel with language production. Language forces the individual's perceptual experience within categories, selecting only some parts of that conglomerate of sensations, perception, and cognition that, on the other hand, keeps substantiating the global non-verbal experience of our relations with the world, most of the times bodily-mediated (see, Werner and Kaplan, 1963). So-called “instinctive” behaviors may then stem from an evaluation of reality based on unconscious procedural memory liable to maintain an active and coherent Self in typical development. What Seganti (1995) refers to as the process of bootstrapping that can be then found in cognitive models of sensorial data, like the Parallel Distributed Processing (e.g., Bucci, 1984; Rumelhart and Mcclelland, 1986; Kihlstrom, 1987), relates to how information associated with the experience of one's body—alive and active—would be recorded in the adult's memory not only in a verbal format, but also in the form of anticipatory sequences of activation states (from the viscera). This idea recalls Damasio's debated proposal of somatic markers (Damasio et al., 1996), according to which signals would arise in bioregulatory processes as related to the body-state structure and to the brain representation of the body leading to, for example, undeliberated inhibition of responses learned previously (Damasio et al., 1996).

Within this scenario, the hard work that narration should undertake stems from the compromise between similarities and oppositions between the sensorial memory of relations—or bodily memory—and the present interactional context with the purpose to adequate one's own involvement to the new relational situation in a continuous, fast and effective manner (see, Seganti, 1995). This would allow individuals to re-establish healthy bridging with their past (see also, Bucci, 1998). Through narratives, it is therefore possible—both in daily life and in therapeutic settings (even though with different modalities and pervasiveness)—to access to unconscious defensive processes and distress-related memories encapsulated and intertwined in our mind and body. Once surfaced, they can be reflected upon and reconstructed by ascribing new meanings and understanding to past events (McAdams, 1993, 1998). Looking specifically at adolescence, the capacity to think about one's own mind, so painstakingly built during infancy, is now put to test and, wherever specific deficiencies are present, they now come to light, pushing the adolescent into a painful and harmful circuit that can result in real psychological problems. A good capacity to mentalize can help the adolescent handle difficult situations avoiding persistent discomfort and maladjustment. In this sense, intervening timely and precociously on risk factors can sensibly impinge upon the way in which young women and men will resolve past situations to face a more serene future. Bodily and representational have to be melded together, both conceptually and with respect to prevention and rehabilitation measures suited to aid adolescents in distress, fruitfully adjoining concepts like internal working models of attachment, the “procedural and sensorial memory of relationships,” narrative as intended by Bruner, and the concept of Stern's “present moment, now” (Stern, 2004) as non-verbal means of psychic transformation.

Lastly, while we here made an attempt to outline the role of the body as a carrier of implicit memories strongly affecting behavior over development—from childhood to adulthood passing through the winding road of adolescence—we would like to ultimately bring forward not only the role of one's own body, but rather of the individual's body in relation to the others' minds and, mostly, to the *others' bodies*. For all the matters here addressed, this is particularly relevant when dealing with adolescence. The need emerges preponderant to reflect on theoretical models, educational and clinical at once, in which one's own body is regarded in relation to the others' bodies as a regulative system of the Self. The selection of our partners, friends, and the relational circle in general is no coincidence. Relationships do not develop only on the basis of a meeting of minds but also—and perhaps primarily and at a deeper implicit level—on recognition and synchronization dynamics, which are in our opinion bodily grounded.

## Author contributions

All authors listed have made a substantial, direct and intellectual contribution to the work, and approved it for publication.

### Conflict of interest statement

The authors declare that the research was conducted in the absence of any commercial or financial relationships that could be construed as a potential conflict of interest.
